# Prioritizing the Compensation Mechanisms for Nurses Working in Emergency Department of Hospital Using Fuzzy DEMATEL Technique: A Survey from Iran

**DOI:** 10.5539/gjhs.v6n2p86

**Published:** 2013-12-01

**Authors:** Jahanara Mamikhani, Shahram Tofighi, Jamil Sadeghifar, Majied Heydari, Vahied Hosseini Jenab

**Affiliations:** 1 Health Management and Economics Research Center, School of Health Management and Information Sciences, Iran University of Medical Sciences, Tehran, Iran; 2 Behin Pooyan Hootan Research Institute, Tehran, Iran; 3 Health Management Research Centre, Baqiyatallah University of Medical Sciences, Tehran, Iran; 4 Hospital Management Research Center, Iran University of Medical Sciences, Tehran, Iran; 5 School of Public Health, Tehran University of Medical Sciences, Tehran, Iran; 6 Natural Disaster Research Institute of Iran, Tehran, Iran

**Keywords:** compensation mechanisms, nurse, emergency department, decision making trial and evaluation laboratory (DEMATEL)

## Abstract

**Aim and Background:**

Nursing professionals are the most important human resources that provide care in the Emergency Departments at hospitals. Therefore appropriate compensation for the services provided by them is considered as a priority. This study aims to identify and prioritize the factors affecting the compensation for services provided by the EDs nurses.

**Methods:**

Twenty four nurses, hospital administrators, local and national health authorities participated in a cross sectional study conducted in 2012. The participants discussed on different compensation mechanisms for nurses’ of EDs, in six groups according to Focus Group Discussion (FGD) technique, resulted in the adopted mechanisms. Opinions of the participants on the mechanisms were obtained via paired matrices using fuzzy logic. Decision Making Trial and Evaluation Laboratory (DEMATEL) technique was used for prioritizing the adopted mechanisms.

**Findings:**

Among the compensation mechanisms for nurses of ED services, both Monthly fixed amounts (9.0382) and increasing the number of vacation days (9.0189) had highest importance. The lowest importance was given to the performance-based payment (8.9897). Monthly fixed amounts, increasing the number of vacation days, decreasing the working hours and performance-based payment were recognized as effective factors. Other mechanisms are prioritized as use of the facilities, increase in emergency tariff, job promotions, non-cash payments, continuing education, and persuasive years.

**Conclusion:**

According to the results, the nurses working in the ED_S_ of the hospitals were more likely to receive non-cash benefits than cash benefits as compensation.

## 1. Introduction

Compensation systems have been substituted for the terms like pay roll management and payment system in the last two decades. This refers to all the payments including cash and non-cash benefits for the employees and managers depending on their performance, the type of organization, conditions of the work environment and characteristics of the job. Compensation systems are an effective strategy to achieve organizational goals and to increase the efficiency of the employees’ performance (Kameli, Darabi, Jafari, & Namjo, 2011). Mechanisms of payment have a significant effect on the performance and efficacy of service providers, and satisfaction of the customers. Therefore, one of the major reforms in the health systems are related to the payment mechanisms (Sæther, 2004).

One of the occupations that have been considered seriously for compensating the services provided in the health systems is nursing. The nurses as the largest group in hospital play an important role in the success of hospital (Hall, 2002). Providing high quality services and having high productivity require adequate number of trained and skilled nurses. Several studies have noted shortage of nurse as an obstacle for achieving the quality of care (McNeese-Smith, 2001; Aiken, Clarke, & Sloane, 2002; Eastaugh, 2002). Different researches have considered the dissatisfaction of nurses as a cause of workforce shortage (Dehghan Nayeri, Nazari, Salsali, & Ahmadi, 2005; Oulton, 2006; Tschannen & Kalisch, 2009). Dehghan Nayeri et al. (2005) indicated that shortage of nurses may lead to increase mistakes, diminish the quality of care, and enhance patients’ dissatisfaction rate.

In Iran, shortage of nurses in hospitals is one of the most important problems. The number of working nurses in Iran is estimated to be one third of the international standard (Sharifi Moghadam, 2010). This condition is a worldwide one, for example as Sæther mentioned in many developing countries there is a great nursing shortage. One of the main reasons for shortage of nurses is the impact of wages on the labor supply (Sæther, 2004).

There is limited available data at the international level in relation to the compensation for the services provided by the nurses and its changing trend. Most of the countries have studied on how the payroll system can be equitable, and have described the differences among different payroll models. In a study conducted in 2009 by the University of Granada in Spain, the econometric techniques were used to determine the amount of wages paid to health related professions. The findings indicate a significant increase in wages for doctors and nurses, better payments for government staff, and a wage gap in favor of men (Salas-Velasco, 2010). In Organization for Economic Cooperation and Development (OECD) countries, between the 2003 and 2009, compensation in the nursing profession has increased more rapidly as compared to other professions (Buchan & Black, 2011). Studies conducted in Norway, The Great Britain and The United States of America reveal that higher wages can reduce the level of displacement of nurses from this profession. However, some evidences suggest that the costs paid for the displacement are higher than the costs when the nurses are paid more. These evidences are limited, related to some specific countries and are constrained with methodological limitations (Bacon, 2011).

Reports of various countries represent the data related to the compensation of services in three ways:

1) The amount of compensation for all nurses’ staff with an average wage is compared among countries. This comparison reveals that people consider the nursing profession as a relatively fetching profession, in terms of financial and social status.
2) The level of compensation in each country is based on the current currency of that country and is calculated on the basis of American dollar. This is used to determine the purchasing power parity (PPP). On this basis, the economic status of the nurses is compared with those in other countries.
3) Average annual growth rate for compensation of services provided by the nursing staff is compared with other staffs that are analyzed during the specific time periods (Bacon, 2011).

Most of the nursing staff is not willing to take the responsibility to work in this department due to the difficulty and stress of the job. The authors’ experiences reveal that there is no strong and powerful incentive to encourage and persuade working in the ED_S_. Furthermore, sometimes working in the ED_S_ is used as a punishment for some nurses, which leads to their break up and displacement from working in this department. Overall, this important department rarely has sufficient, capable and highly motivated staff for providing appropriate diagnostic and vital on time services. In this situation, long waiting time, low quality and incomplete treatment are expectable. The usual strategies that can decrease injustice and the sense of disenchantment in nurses working in ED_S_ are taking the higher rates of compensation, performance-based pay, payment for work difficultness and providing financial support for its implementation (Sæther, 2005). In this sense, the present study utilizes the expert opinion to prioritize the compensation mechanisms of the nurses working in ED_S_ of the hospitals in Iran, so that the results obtained can be a valuable evidence for the policy makers and provides appropriate guidance for the executives.

## 2. Methods

Twenty four nurses, hospital administrators, local and national health authorities participated in a cross sectional survey conducted in 2012. The participants discussed on different compensation mechanisms for nurses’ ED services, in six different groups, comprising homogenous participants, according to FGD technique, as a following:

1) Heads of nursing offices in Treatment Deputy of the selected medical universities.
2) Administrators of the selected hospitals.
3) Nurses with experience working in ED of hospital from a deprived city of the selected province.
4) Nurses with experience working in ED of hospital with less than 150 beds in the selected provinces.
5) Nurses with experience working in ED of hospital in one of the major referral hospital of the capital of selected provinces.
6) Expert nurses working in National Nursing Council, Iranian Nursing Association, Nursing Office of the Ministry of Health, and Emergency Office.

Data was collected using the questionnaire developed by the researchers. The questionnaire contained 10 factors including: monthly fixed amounts, increase in vacation days, priority in use of the hospital facilities, decrease in working hours, increase in tariffs for ED, job promotions, non-cash payments, higher education grants, persuasive years, and performance-based. Opinions of the participants on the mechanisms were obtained via paired matrices. Weight of factors and interrelationship among factors were determined by DEMATEL technique, using MATLAB software. DEMATEL method, a method for decision making, was originally developed in order to study the complex problematic issues. It has been widely accepted as one of the best tools to solve the cause and effect relationship among the evaluation criteria (Chiu, Chen, Tzeng, & Shyu, 2006; Liou, Tzeng, & Chang, 2007; Tzeng, Chiang, & Li, 2007; Wu & Lee, 2007). This method is applied to derive interrelationship among factors (Lin & Tzeng, 2009). The steps of DEMATEL are as follow:

### 2.1 Preparing of Fuzzy Direct Relations Matrix

Paired comparisons were performed between the main mechanisms and effect of the factor *i* in a row on factor *j* in a column was obtained. Fuzzy positive numbers that have been used for these comparisons are shown in Table 1.

**Table 1:** Expression variables and the corresponding fuzzy numbers

values of the expression Scales	Triangular fuzzy numbers
Very Large effect	(0.75, 1.0, 1.0)
Large effect	(0.5, 0.75, 1)
Low effect	(0.25, 0.5, 0.75)
Very low effect	(0, 0.25, 0.5)
No effect	(0, 0, 0.25)

### 2.2 Preparing Normalized Matrix of Direct Relations Matrix

In this stage, CFCS method that introduced by Aprikovich and Tsang was used. For normalized direct relations matrix, following relations were used:

Triangular fuzzy number was considered as 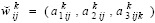 and in which K is the expert person.

### 2.2.1 Normalization


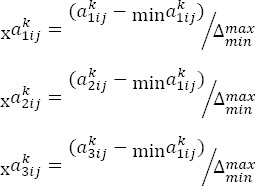



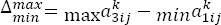


### 2.2.2 Calculation of the Left Normal (Is) and Right (Rs) Values


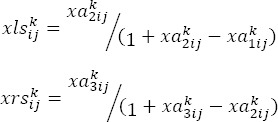


### 2.2.3 Calculation of the Final and Absolute Normalized Value


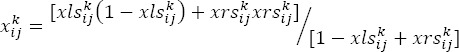


### 2.2.4 Calculation of the Absolute Values


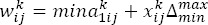


### 2.2.5 Combination of Absolute Values


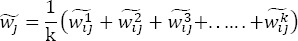


## 2.3 Formation of Overall Relations Matrix

After obtaining the matrix of the combined opinions of the experts (w), by the following equation Matrix X is calculated.


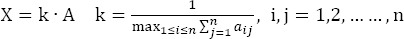


The matrix of the overall relations is calculated by the following equation where *I* is the mentioned matrix.


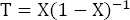


## 2.4 Preparing the Diagram of Effector and Effect Relations

Total components of rows and columns of the matrix T were named as D and R vectors, respectively. D represents the sum of the direct and indirect effects of the factor i on others. While R represents the sum of the direct and indirect effects of others. If D+R is large for a particular factor, it shows that this factor has more interactions with other factors. Positive D-R for a factor represents the causal factor and shows that the value i has effects on others, and negative value for D-R reveals that the i is affected by other factors. D and R are also obtained from the following equations:


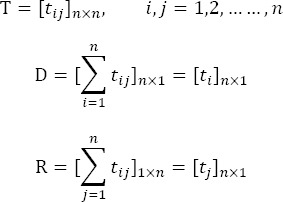


Finally causal diagram through drawn of points with coordinates (D+R, D-R) for each factor is obtained by a Cartesian coordinate system.

## 3. Findings

**Figure 1:**
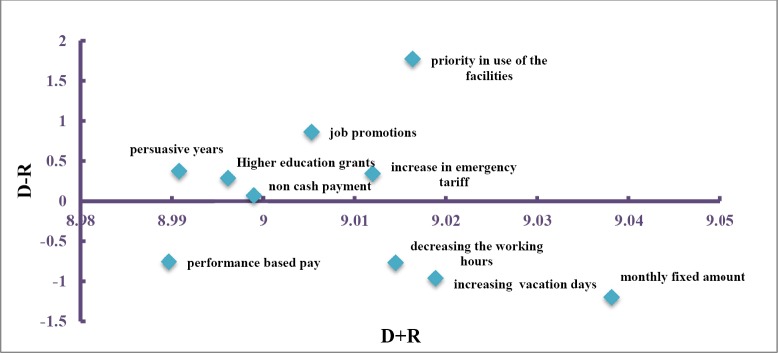
Diagram of effectors among compensation mechanisms for nurses working in ED_S_

The result of the paired comparisons for importance of the different compensation mechanisms in the view of nurses working in the ED_S_, is shown in Tables 2 to 5. According to the Table 4 and Figure 1, monthly fixed amounts, increase in vacation days, priority in use of the hospital facilities, decrease in working hours, increase in tariffs for emergency ward, job promotions, non-cash payments, higher education grants, persuasive years, and performance-based payments have the greatest effects, respectively. Negative D-R shows that the monthly fixed amounts, increased vacation, reduced working hours and performance-based payment are more important factors.

**Table 2:** Matrix of primary direct relationship among importance of compensation mechanisms in view of nurses working in ED_S_

	decreasing the working hours	increasing vacation days	increase in emergency services’ tariffs	monthly fixed amount	non cash payments	Priority in use of the facilities	job promotions	Performance- based payments	persuasive years	Higher education grants
decreasing the working hours	0	0.4631	0.5	0.5122	0.4754	0.3526	0.4263	0.4877	0.4385	0.4263
increasing vacation days	0.5368	0	0.3771	0.5368	0.4508	0.3649	0.3894	0.5122	0.4017	0.4263
increase in emergency tariff	0.5	0.6228	0	0.5736	0.5368	0.4508	0.4877	0.5491	0.4754	0.4877
monthly fixed amount	0.4877	0.4631	0.4263	0	0.4017	0.3649	0.4263	0.3526	0.4631	0.4631
non cash payment	0.5245	0.5491	0.4631	0.5982	0	0.4017	0.4877	0.5491	0.5	0.4631
priority in use of the facilities	0.6473	0.6350	0.5491	0.6350	0.5982	0	0.5859	0.6105	0.5982	0.5982
job promotions	0.5736	0.6105	0.5122	0.5736	0.5122	0.4140	0	0.5859	0.6228	0.5614
performance based pay	0.5122	0.5	0.4508	0.6473	0.4508	0.3894	0.4140	0	0.3649	0.3649
persuasive years	0.5614	0.6105	0.5245	0.5368	0.5	0.4017	0.3771	0.6350	0	0.5614
Higher education grants	0.5736	0.5859	0.5122	0.5368	0.5368	0.4017	0.4385	0.6350	0.4385	0

**Table 3:** Normalized matrix of direct relationship among importance of compensation mechanisms in view of nurses working in ED_S_

	decreasing the working hours	increasing vacation days	increase in emergency services’ tariffs	monthly fixed amount	non cash payments	Priority in use of the facilities	job promotions	Performance- based payments	persuasive years	Higher education grants
decreasing the working hours	0.0000	0.0849	0.0916	0.0939	0.0871	0.0646	0.0781	0.0894	0.0804	0.0781
increasing vacation days	0.0984	0.0000	0.0691	0.0984	0.0826	0.0669	0.0714	0.0939	0.0736	0.0781
increase in emergency tariff	0.0916	0.1141	0.0000	0.1051	0.0984	0.0826	0.0894	0.1006	0.0871	0.0894
monthly fixed amount	0.0894	0.0849	0.0781	0.0000	0.0736	0.0669	0.0781	0.0646	0.0849	0.0849
non cash payment	0.0961	0.1006	0.0849	0.1096	0.0000	0.0736	0.0894	0.1006	0.0916	0.0849
priority in use of the facilities	0.1186	0.1164	0.1006	0.1164	0.1096	0.0000	0.1074	0.1119	0.1096	0.1096
job promotions	0.1051	0.1119	0.0939	0.1051	0.0939	0.0759	0.0000	0.1074	0.1141	0.1029
performance based pay	0.0939	0.0916	0.0826	0.1186	0.0826	0.0714	0.0759	0.0000	0.0669	0.0669
persuasive years	0.1029	0.1119	0.0961	0.0984	0.0916	0.0736	0.0691	0.1164	0.0000	0.1029
Higher education grants	0.1051	0.1074	0.0939	0.0984	0.0984	0.0736	0.0804	0.1164	0.0804	0.0000

**Table 4:** Matrix of the overall relationship among importance of compensation mechanisms in view of nurses working in ED_S_

	decreasing the working hours	increasing vacation days	increase in emergency services’ tariffs	monthly fixed amount	non cash payments	Priority in use of the facilities	job promotions	Performance- based payments	persuasive years	Higher education grants
decreasing the working hours	0.3727	0.4585	0.4142	0.4762	0.4202	0.3371	0.3827	0.4532	0.4025	0.4042
increasing vacation days	0.4538	0.371	0.3875	0.471	0.4084	0.3325	0.3697	0.4481	0.389	0.3963
increase in emergency tariff	0.507	0.5341	0.3746	0.5381	0.4755	0.3896	0.434	0.5126	0.4524	0.4585
monthly fixed amount	0.4367	0.44	0.3867	0.3708	0.3922	0.3254	0.3673	0.4144	0.3905	0.3941
non cash payment	0.4977	0.5095	0.4415	0.5283	0.3739	0.3723	0.4231	0.4995	0.4448	0.4432
priority in use of the facilities	0.5944	0.6022	0.5239	0.6152	0.5442	0.3614	0.5031	0.5869	0.5286	0.5333
job promotions	0.5415	0.556	0.4813	0.5622	0.4929	0.4011	0.3709	0.5418	0.4953	0.4906
performance based pay	0.4578	0.463	0.4058	0.4962	0.4155	0.3422	0.3804	0.3695	0.3906	0.394
persuasive years	0.5167	0.5325	0.4627	0.533	0.4701	0.3822	0.417	0.5264	0.3718	0.4697
Higher education grants	0.5147	0.5248	0.4574	0.5291	0.4723	0.3794	0.4235	0.5223	0.4432	0.3728

**Table 5:** Important compensation mechanisms in view of nurses working in ED_S_

	D	R	D+R	D-R
monthly fixed amounts	3.9181	5.1201	9.0382	-1.202
increasing vacation days	4.0273	4.9916	9.0189	-0.9643
priority in use of the facilities	5.3932	3.6232	9.0164	1.77
decreasing the working hours	4.1215	4.893	9.0145	-0.7715
increase in emergency tariff	4.6764	4.3356	9.012	0.3408
job promotions	4.9336	4.0717	9.0053	0.8619
non cash payment	4.5338	4.4652	8.999	0.0686
Higher education grants	4.6395	4.3567	8.9962	0.2828
persuasive years	4.6821	4.3087	8.9908	0.3734
performance based pay	4.115	4.8747	8.9897	-0.7597

## 4. Discussion and Conclusion

In the present study, the views of nurses working in ED_S_ of hospitals, for preferred compensation mechanisms through gathering opinions of experts, are determined. Here, most of the participants, who worked in a small hospital, or nurses working in the ED of referral hospitals or the administrative office of the ministry of health, were more willing to receive non-cash payments, particularly the benefits that create a feeling of being special in nurses such as welfare benefits, than cash payments. In the previous studies, the non-cash benefits were highlighted and emphasized by the nurses and authors. Their findings completely match with present study. In some developed countries that nurses tend to have higher welfare benefits because the payments received by them don’t differ much from the doctors. But the wage’s gap in Iran is very large. It should be explored why the nurses in Iran have lower willingness to get paid more.

In Siqueira et al’s (2012) study about factors affecting job satisfaction among nurses, compensation factor was seen to be one of the least priority factors for job satisfaction, although this factor was an important reason for dissatisfaction. Nursing managers gave less importance while clinical nurses gave more importance to compensation (Siqueira & Kurcgant, 2012). Paiva et al. (2011) carried out a study about the job satisfaction of nurses working in a home care by using the professional satisfaction index (PSI). Based on their study, the compensation factor was the third priority after autonomy and interactive factors in relation to the job satisfaction. Joolaee et al. (2006) conducted a study on the nursing students of the Tehran University of Medical Sciences, 69% of the samples were willing to leave the nursing field. Lack of positive social base, gap between of the perceptions and expectations, negative attitude of the medical team towards their profession, inappropriate clinical working environment, status gap between the doctors and nurses and low wages of nurses were the main reasons for leaving the nursing profession in this research.

One of the issues suggested for the compensation of services is that the cultural and psychological features need to be surveyed. Psychological needs of nurses are suggested in this research. This study has potential implications to nursing policies/practices. Healthcare managers in local and national levels can provide appropriate infrastructure for enhancing nurse’s motivation, satisfaction and their productivity. This can be conducted through emphasizing on more important mechanisms of nurse’s compensation, diminishing inequity between nursing profession and other professions. The best important strengths in this study are related to interesting topic and data gathering methods. An insufficient literature particularly in national level for comparison current findings with them is a key limitation in this study.
